# Affordability, negative experiences, perceived racism, and health care system distrust among black American women aged 45 and over

**DOI:** 10.3934/publichealth.2024053

**Published:** 2024-09-26

**Authors:** Jacqueline Wiltshire, Carla Jackie Sampson, Echu Liu, Myra Michelle DeBose, Paul I Musey, Keith Elder

**Affiliations:** 1 Indiana University Fairbanks School of Public Health, Indianapolis, IN USA; 2 Robert F. Wagner Graduate School of Public Service, New York University, NY USA; 3 College for Public Health and Social Justice, Saint Louis University, MO USA; 4 College of Nursing and School of Allied Health, Northwestern State University, LA USA; 5 Indiana University School of Medicine, Indianapolis, IN USA; 6 Saint Xavier University, Chicago, IL USA

**Keywords:** healthcare affordability, negative experiences, racism, healthcare system, distrust, African American women

## Abstract

Black Americans (AA) face a confluence of challenges when seeking care including unaffordable costs, negative experiences with providers, racism, and distrust in the healthcare system. This study utilized linear regressions and mediation analysis to explore the interconnectedness of these challenges within a community-based sample of 313 AA women aged 45 and older. Approximately 23% of participants reported affordability problems, while 44% had a negative experience with a provider. In the initial linear regression model excluding perceived racism, higher levels of distrust were observed among women reporting affordability problems (*β* = 2.66; *p* = 0.003) or negative experiences with a healthcare provider (*β* = 3.02; *p* = <0.001). However, upon including perceived racism in the model, it emerged as a significant predictor of distrust (*β* = 0.81; *p* = < 0.001), attenuating the relationships between affordability and distrust (*β* = 1.74; *p* = 0.030) and negative experience with a provider and distrust (*β* = 1.79; *p* = 0.009). Mediation analysis indicated that perceived racism mediated approximately 35% and 41% of the relationships between affordability and distrust and negative experience with a provider and distrust, respectively. These findings underscore the critical imperative of addressing racism in the efforts to mitigate racial disparities in healthcare. Future research should explore the applicability of these findings to other marginalized populations.

## 1. Introduction

Americans' distrust in the healthcare system, which can be broadly defined as harboring negative expectations regarding the conduct of healthcare providers and systems [1], is on the rise [2], with notable links to escalating costs of care and affordability issues [3]. Americans, especially those of lower-income and diverse racial/ethnic backgrounds, experience problems paying medical bills, delay medical treatment, incur substantial medical debt, and even file bankruptcy due to unaffordable healthcare costs [4]–[6]. This intertwining of healthcare system distrust and affordability problems is associated with deteriorating physician–patient relationships and adverse health outcomes [7],[8]. Moreover, these relationships may exhibit a reciprocal nature. Individuals perceiving healthcare as unaffordable may forgo seeking treatment, thereby exacerbating their health issues and reinforcing their distrust in the healthcare system. However, existing research often fails to address how these factors uniquely impact diverse populations.

Black Americans are well-documented as having one of the highest levels of distrust in the healthcare system [9],[10]. This distrust is often attributed to a history of racism, discrimination, mistreatment, or negative experiences in the healthcare system. Throughout the late nineteenth and early twentieth centuries, Black Americans were mistreated and experimented on by medical institutions [11]–[13]. Examples among Black women include experimental surgeries without anesthesia, forced sterilization, and use of bodily tissue without consent or permission [14]. Pseudoscientific theories about biological differences between Black and White people have been used to justify racial discrimination and unequal treatment in healthcare [12].

Discrimination significantly shapes the current healthcare experiences of Black Americans. Many perceive that they receive poorer quality healthcare and are treated with less respect than White Americans [15]. They have also reported experiencing multiple negative encounters with healthcare providers [16]. A 2020 national survey exploring Black Americans' views and experiences with racism and discrimination within the context of the COVID-19 pandemic [15] found that Black Americans, compared to White Americans, had less trust in doctors, hospitals, and the healthcare system to act in the best interest of their communities. Martin and colleagues [17] found that present-day negative experiences in the healthcare system were more salient to Black Americans' distrust than knowledge of past abuses. Understanding the impact of negative experiences is especially important to address Black Americans' distrust in the healthcare system.

Black Americans, particularly older adults, tend to disproportionately experience healthcare affordability problems and related consequences [6]. In 2022, approximately 60% of Black adults reported difficulty affording healthcare costs compared with 39% of White adults [18]. Compared with White workers, Black workers tend to have limited access to quality employment opportunities and employer-sponsored health insurance, which leaves them either uninsured or underinsured, with lower wages or income to pay for healthcare services [19]. Studies also show that Black Americans are more likely to experience debt collection and discriminatory practices in debt collection, including threats of legal action, higher interest rates, and harsher penalties resulting from unaffordable healthcare services [19].

Black women, who comprise one in every seven women in the US, are disproportionately represented among people living in poverty and employed in low-wage jobs that lack benefits and opportunities for wealth accretion [20]. Essentially, Black women tend to face intersecting factors of race- and gender-based discrimination that impact their overall well-being [20]. Moreover, older Black women, those aged 45 and older, have an additional marginalized identity of age along with the health challenges that come with midlife and aging, which tend to further negatively shape their healthcare experiences [20]. Few studies have focused on healthcare affordability and the unique experiences, including racism, of older Black women.

There is great emphasis on improving healthcare affordability [21] but a reluctance to recognize racism as a root cause of racial health disparities [22]. Many healthcare organizations have made commitments to address racism and racial/ethnic disparities in healthcare but do not explicitly state how they will address racism [23],[24]. Black Americans perceive racism as a bigger problem (63%) to their well-being than affordability of care (47%) [25]. Research illustrating the magnitude of racism's impact on almost every aspect of the Black healthcare experience, as this study attempts to show, could lead to explicit interventions to address the root cause of health inequalities [26].

While the literature extensively documents the relationship between racism and Black Americans' distrust in the healthcare system, the relationships among healthcare affordability, racism, and distrust are less clear. Understanding the mechanism underlying these relationships is important in addressing health disparities. Although distrust is critically linked to health outcomes, there is a surprising paucity of research on healthcare distrust among older Black American women, who have more interactions with the healthcare system than other Black American subgroups. Black American women constitute an expanding part of the older and sickest population in the United States [27]. They are particularly vulnerable to healthcare affordability problems because they tend to have higher out-of-pocket expenses and fewer financial resources than their White counterparts [6]. In this study, we examine the relationships between affordability, negative experiences with healthcare providers, perceived racism, and healthcare system distrust among older Black women. We also assess the extent to which perceived racism explains the relationships between affordability, negative experiences, and healthcare system distrust.

## 2. Theoretical framework

Examining healthcare affordability among older African American women requires insight from various intersecting frameworks or theories, including Feagin's theory of systematic racism, the life course perspective, and cumulative inequality theory [13],[28],[29]. Feagin's theory posits that racism perpetuates inequalities across every aspect of Black American life, which would include access to and affordability of care. According to Feagin, racism is present not just in overt acts by individuals but also in the everyday social, economic, and political structures and institutions that shape American lives. He contends that these systems were designed to benefit certain racial groups, primarily White, while disadvantaging others, such as African Americans. These “racialized social systems” including education, housing, employment, and healthcare, are an interconnected web of institutions and practices that perpetuate racial inequality [13]. Today, as in the past, racial oppression is not just a surface-level feature of society, but rather it pervades, permeates, and interconnects all major social groups, networks, and institutions across society. These institutions and systems support a caste system that subjugates Black Americans and has persisted in policies and practices for four centuries [30]. Prior research shows that many African Americans believe that systemic racism affects every aspect of their lives [15],[31]. Systemic racism has resulted in limited access to resources and economic opportunities for African Americans, contributing to disparities in areas important to this study, such as wealth accretion, income, and insurance coverage.

Within Feagin's theory of systemic racism, is embedded the concept of “intersectionality”, which underscores how multiple social identities (such as race, gender, gender identity, and age) intersect to differentially shape experiences of discrimination and the cumulative disadvantage of Black American women [32],[33]. Bearing at least two marginalized identities, Black American women are at a double jeopardy disadvantage. Elliot et al. [34] demonstrated that at this intersection, Black women experience these challenges daily, complicated by the stigma of needing or seeking welfare support. Moreover, gendered societal norms, expectations, and biases can exacerbate disparities, as women tend to have multiple roles (e.g., mother, worker, homemaker, and caregiver) and be in lower-paying jobs, which impacts their ability to access and afford care [35].

Social, economic, and political factors can operate at all stages of human development (from birth to death) to influence health or set the foundation for health in later life [28]. The effects of these factors can also accumulate over time to impact health outcomes [28],[36]. According to the cumulative model of the life course perspective, beneficial or detrimental effects of these factors can accumulate over time to produce health benefits or detriments in later life. Studies have documented the gradual deterioration of health or accelerated aging from constant exposure to adverse social, economic, political, and biological factors [28]. African Americans are more likely than their White counterparts to experience the effect of cumulative inequalities in later life [28],[37]. African Americans also have less wealth than White Americans [38] and, consequently, less ability to avoid medical bill problems in later life.

It is important to study healthcare affordability and the role of racism among older African American women as it reflects the historically social, economic, and political disadvantage that is associated with well-being [39]. There are strong theoretical reasons to expect racism to explain the relationship between healthcare affordability and healthcare system distrust, given the historical and structural social, economic, and political structures and institutions that shape American lives and health outcomes [40]. This study aims to fill that gap by exploring the relationships between affordability, negative experiences, and perceived racism. The need for studies on these relationships has increased because the social construct of race has traditionally been used to categorize data rather than to explain research hypotheses. This approach often overlooks the complex interactions between race and other sociodemographic factors such as educational attainment, employment status, income, and wealth in health. Consequently, this approach effectively ignores the role of racism in driving health inequities [41],[42].

## 3. Materials and methods

### 3.1. Study design and sample

Data for this study was collected using self-reported surveys—administered via laptops—in a convenience sample of 313 Black American women aged 45 and older who lived in Madison, Wisconsin between 2008 and 2010. The eligibility criteria also included the ability to provide consent. Given the history of exploitation and mistreatment in medical research, recruiting Black Americans for health studies requires culturally sensitive and community-focused approaches [43]. Therefore, women were recruited through various community avenues, including churches, community health centers, hair salons, health fairs, community events, senior centers and housing establishments, and advertisements targeted at the Black community. Women were also recruited through referral by other study participants. While these community-based recruitment methods may not be representative of all Black Americans, these methods successfully recruit Black Americans into health studies [43]. The sample size was determined by requiring *α* (the probability of a type I error) to be 5% and power (1-type II error probability; 1-*β*) to be 80% [44]. Older and disabled women were surveyed in their homes (*n* = 40) and assisted with the reading of the survey and laptop technology when needed. Participants received a $20 gift certificate for completing the surveys. This study was approved by the University of Wisconsin-Madison Social and Behavioral Science Institutional Review Board.

### 3.2. Measures

Healthcare system distrust is a complex concept that encompasses aspects of fidelity (i.e., promoting patient's interests), competence (interpersonal and technical skills), honesty, confidentiality, compassion, dependability, and communication [7],[45],[46]. This 10-item distrust scale (see Table S1,) was developed and validated by Rose and colleagues [46] and includes two items on honesty (e.g., Medical experiments can be done on me without my knowing about it), two on confidentiality (e.g., My medical records are kept private), two on competence (e.g., People die every day because of mistakes by the healthcare system), and two on fidelity (e.g., The healthcare system puts my medical needs above all other considerations when treating my medical problems). The responses on these items ranged from 1 (“strongly disagree”) to 5 (“strongly agree”). Please refer to Table S1, for the complete list of items and responses on the distrust scale.

Perceived racism in the healthcare system was assessed with 4 items developed and validated by the Cardiac Access Longitudinal study [9]. This four-item index measures the experience of White racism by people of color (e.g., Doctors treat African Americans and White people the same). The response format ranged from 1 (“strongly agree”) to 5 (“strongly disagree”). Please refer to Table S2, for the complete list of items and responses on the scales/indexes.

Healthcare affordability and negative experiences with a provider were assessed with the following questions: Was there a time in the past 12 months when you needed to see a doctor but could not because of costs? Have you had a negative experience with a healthcare provider? Responses for these questions were coded as yes/no.

Control variables included in the analysis were age (45–64, 65+), marital status (married, unmarried), education level (less than high school, high school, some college, college and above), income level (<$25,000, $25,000–$49,999, ≥$50,000), insurance status (insured, uninsured), perceived health status (poor/fair, good, and very good/excellent), and having a usual healthcare provider (yes/no). These variables were chosen based on existing literature, which suggests that distrust and perceived racism in the healthcare system are linked to patient sociodemographic characteristics, health status, access to care, and medical care factors [47],[48].

### 3.3. Data analysis

Descriptive analyses (frequency, percentage, mean, and standard deviation) were conducted on all study variables. Pearson's correlation was utilized to examine the associations between healthcare affordability, negative experience with a provider, perceived racism, and healthcare system distrust. Linear regression analyses, adjusted for control variables, were conducted to examine the relationships between affordability, negative experiences with a provider, and distrust in the healthcare system. Beta coefficients (*β*), standard errors (*SE*), and p-values from the regression models were used to characterize associations. *R^2^* values were used to indicate the proportion of variance of the dependent variable (i.e., distrust) explained by the independent variables in the regression models.

To investigate whether perceived racism mediates the relationships between affordability, negative experience with provider, and healthcare system distrust, we conducted mediation analysis using the structural equation modeling (SEM) framework. SEM enables simultaneous estimation of regression models and hypothesis testing regarding variable relationships [49]. This study employs an SEM model encompassing affordability, negative experience with a healthcare provider, and distrust variables to elucidate their interrelations. Our mediation analysis adopts a modified Baron and Kenny approach, supplemented by a bootstrap test of indirect effects proposed by Zhao and colleagues [50].

**Figure 1.**
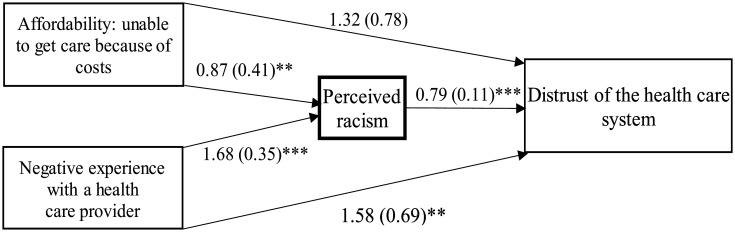
The mediated effect of perceived racism on the relationships between unable to get care because of costs, negative experience with health care provider, and health care system distrust (*N* = 313). Note: ** indicates significance at the 5% level, and *** indicates significance at the 1% level.

We employed Stata's built-in ‘sem’ command followed by the ‘medsem’ post-estimation command to assess our mediational hypotheses; specifically, whether affordability and negative experience with a provider influence healthcare system distrust through both indirect (mediated) and direct pathways. The total contribution of each path was computed as the product of the path coefficients. The mediated proportion was determined as the ratio of the contribution of a specific indirect path to the total contribution of all paths. The paths for affordability (i.e., affordability to perceived racism, perceived racism to distrust, affordability to distrust) and negative experience with a provider (i.e., negative experience to perceived racism, perceived racism to distrust, negative experience to distrust) are depicted in the mediation diagram in Figure 1. To evaluate the significance of the indirect effect of perceived racism on the relationships between healthcare affordability, negative experience with a provider, and healthcare system distrust, we employed the Monte Carlo test, which adjusts for non-normality in the standard errors of the indirect effects [51]. All data analyses were performed using STATA software (Version 17.0). A *p*-value < 0.05 was considered statistically significant for all tests.

## 4. Results

### 4.1. Descriptive and bivariate analyses

Table 1 displays the characteristics of the study sample comprising 313 individuals. Approximately 23% of participants reported affordability problems, while 44% reported a negative experience with a provider. The mean score on the healthcare system distrust scale stood at 28.9 out of a possible 50 points, with a mean perceived racism score of 14.1 out of 20. The Cronbach's alphas for the distrust and perceived racism scales were 0.78 and 0.70, respectively. Pearson correlations (Table S3,) identified positive correlations between distrust in the healthcare system and healthcare affordability (*r* = 0.14, *p* < 0.05), negative experience with a provider (*r* = 0.23, *p* < 0.001), and perceived racism (*r* = 0.43, *p* < 0.001). Affordability (*r* = 0.13, *p* < 0.05) and negative experience with a provider (*r* = 0.27, *p* < 0.001) were significantly positively correlated with perceived racism in the healthcare system. There was no correlation between healthcare affordability and negative experience with a provider (*r* = 0.05, *p* > 0.05).

The *p*-values derived from *F* statistics, as reported in Table 1, indicate that the affordability of healthcare and the feeling of mistreatment when receiving healthcare help explain the variations in perceived racism and distrust in the healthcare system, respectively, when other factors are held constant. While the isolated insignificance implied by the substantial *p*-values holds for several other covariates, their inclusion in the regression analysis still contributes to a more meaningful body of research for this study. Table 2 displays the correlations between all covariates. In general, these variables are not strongly related to each other, meaning that the chance of having unreliable estimates with their inclusion in the regression analysis is insignificant. However, it is interesting to observe that skipping needed care due to concerns about affordability is positively correlated with having a usual healthcare provider while being adversely related to having insurance. Additionally, age is inversely correlated with insured status, and being insured is positively associated with health status.

### 4.2. Linear regression analyses

The results from the linear regression models, which investigate the relationship between affordability, negative experience with a provider, perceived racism, and distrust of the healthcare system, are summarized in Table 3. The variance inflation factor (VIF) for each independent variable (values not displayed) is below 3, with a mean of 1.65, indicating no significant multicollinearity issues in our regressions. In Model 1, perceived racism was found to be higher among women who reported healthcare affordability problems (*β* = 1.14; *p* = 0.014) compared with those without such problems. Similarly, perceived racism was 1.5 points higher among women reporting a negative experience with a provider (*β* = 1.51; *p* = 0.000) compared with those who did not report having a negative experience with a provider. Furthermore, women aged 65 and above perceived less racism in healthcare compared to those aged 45–64 (*β* = −0.88; *p* = 0.041).

**Table 1.** Characteristics of the study sample (*N* = 313).

Project	*Mean*/standard deviation (*SD*)	*n* (%)	*p*-value^c^
Perceived racism^a^	14.1 (3.2)		
Healthcare system distrust^b^	28.9 (6.5)		
Affordability: unable to get needed care because of costs			0.02, 0.01
Yes		240 (76.68)	
No		73 (23.32)	
Negative experience with a provider			0.00, 0.00
Yes		176 (56.23)	
No		137 (43.77)	
Age			0.00, 0.77
45–64		229 (73.16)	
65+		84 (26.84)	
Married			0.37, 0.45
Yes		95 (30.35)	
No		218 (69.65)	
Education			0.02, 0.91
< High school		57 (18.21)	
High school		72 (23.00)	
Some college		97 (30.99)	
College +		87 (27.80)	
Income			0.01, 0.55
< $25,000		143 (45.69)	
$25,000–$49,000		96 (30.67)	
$50, 000 or more		74 (23.64)	
Insured			0.88, 0.38
Yes		263 (84.03)	
No		50 (15.97)	
Usual healthcare provider			0.78, 0.67
Yes		293 (93.61)	
No		20 (6.39)	
Self-reported health			0.34, 0.67
Poor/fair		99 (31.63)	
Good		137 (43.77)	
Very good/excellent		77 (24.60)	

Note: a. The maximum score for distrust in the healthcare system is 50. Higher scores indicate greater distrust. b. The maximum score for perceived racism is 20. Higher scores indicate greater perceived racism. c. The first *p*-value is derived from the test of association between the variable and racism score, while the second *p*-value originates from the test of association between the variable and distrust score.

Models 2 and 3 illustrate that affordability, negative experience with a provider, and perceived racism were the only significant predictors of distrust in the healthcare system. In Model 2 (without adjusting for perceived racism), the score for distrust in the healthcare system was approximately 2.7 points higher for women facing affordability problems (*β* = 2.66; *p* = 0.003) and three times higher for those who had a negative experience with a provider (*β* = 3.02; *p* = 0.000) than those who did not. Model 3 reveals that perceived racism within the healthcare system was a significant predictor of healthcare system distrust (*β* = 0.81; *p* = 0.000), also attenuating the associations between affordability (*β* = 1.74; *p* = 0.030) and negative experience with a provider (*β* = 1.78; *p* = 0.009). The *R^2^* in Model 2 (without adjusting for perceived racism) increased from 0.09 to 0.23 with the inclusion of perceived racism (Model 3), indicating a substantial contribution to explaining variations in distrust scores. In these two specifications, sociodemographic variables, health status variables, and access to care variables did not emerge as significant predictors of distrust in the healthcare system.

**Table 2.** Correlation between independent variables (*N* = 313).

Project	Affordability: unable to get needed care because of costs	Negative experience with a provider	Age	Married	Education	Income	Insured	Usual healthcare provider	Self-reported health
Affordability: unable to get needed care because of costs	1.00								
Negative experience with a provider	0.05	1.00							
Age	0.10	0.08	1.00						
Married	−0.08	−0.06	−0.18**	1.00					
Education	0.15**	−0.19***	−0.27***	0.09	1.00				
Income	0.20***	−0.12**	−0.20***	0.34**	0.54***	1.00			
Insured	−0.34***	−0.02	−0.21***	−0.04	−0.01	−0.20***	1.00		
Usual healthcare provider	0.23***	0.03	0.07	0.09	0.03	0.10	−0.17**	1.00	
Self-reported health	0.04	−0.01	−0.17**	0.17**	0.38***	0.36***	0.02	−0.01	1.00

Note: ** indicates significance at the 5% level, and *** indicates significance at the 1% level.

### 4.3. Mediation analyses

Figure 1 displays the path coefficients and their significance level resulting from the SEM analysis, encompassing variables measuring affordability, negative experience with a provider, perceived racism, and healthcare system distrust while excluding confounding factors. Regarding the affordability–distrust relationship, the indirect effect of affordability was estimated at 0.69 with a *p*-value of 0.040 using Monte Carlo standard errors, without considering any confounding factors, as shown in Table 4. In contrast, its direct effect was estimated at 1.32 with a *p*-value of 0.105 in this context. The calculated mediation proportion (i.e., indirect effect/total effect) suggests that approximately 34% (0.69/2.01) of the association between affordability and healthcare system distrust operates through perceived racism. In a fully adjusted model (i.e., with all control variables included), approximately 35% (0.93/2.67) of the affordability–distrust relationship operates through perceived racism, as indicated by estimates reported in Table 4.

Perceived racism also serves as a mediator in the relationship between a negative experience with a provider and healthcare system distrust. The indirect effect of a negative experience with a provider was estimated at 1.32 with a *p*-value of <0.001 using Monte Carlo standard errors when no confounding variables are considered, as detailed in Table 4. The direct effect under these conditions was estimated at 1.58, with a *p*-value of 0.024. These estimates suggest that approximately 45% (1.32/2.91) of the association between negative experience with a provider and healthcare system distrust operates through perceived racism. In a fully adjusted model, approximately 41% (1.23/3.02) of the negative experience with a provider–distrust relationship is mediated through perceived racism, based on the estimates provided in Table 4.

**Table 3.** Regression coefficient estimates: exploring the association between independent variables and perceived racism in healthcare and distrust in the healthcare system scores (*N* = 313).

Variables	Model 1: The dependent variable is the score for perceived racism in healthcare	Model 2: The dependent variable is the score for healthcare system distrust	Model 3: Healthcare system distrust score as the dependent variable, incorporating perceived racism score as an independent variable
Affordability: unable to get needed care because of costs			
Yes	1.14 (0.46)**	2.66 (0.88)***	1.74 (0.80)**
No (reference)			
Negative experience with a provider			
Yes	1.51 (0.35)***	3.02 (0.75)***	1.79 (0.68)***
No (reference)			
Age			
45–64 (reference)			
65+	−0.88 (0.43)**	0.39 (0.94)	1.10 (0.83)
Married			
Yes	−0.21 (0.44)	−0.90 (0.92)	−0.73 (0.85)
No (reference)			
Education			
< High school	−0.39 (0.70)	−0.38 (1.43)	−0.06 (1.29)
High school	−0.24 (0.56)	−0.19 (1.22)	0.01(1.11)
Some college	−0.27 (0.49)	−0.65 (1.08)	−0.44 (0.95)
College + (reference)			
Income			
< $25,000	−1.09 (0.56)	−0.33 (1.35)	0.55 (1.22)
$25,000–$49,000	−0.18 (0.55)	0.93 (1.22)	1.08 (1.08)
$50,000 or more (reference)			
Insured			
Yes (reference)			
No	−0.34 (0.50)	−1.76 (1.01)	−1.48 (0.98)
Usual healthcare provider			
Yes (reference)			
No	−0.38 (0.65)	−0.41 (1.24)	−0.09 (1.27)
Self-reported health			
Poor/fair	0.11 (0.54)	0.74 (1.10)	0.65 (1.03)
Good	0.47 (0.48)	0.47 (1.01)	0.09 (0.96)
Very good/excellent (reference)			
Score for perceived racism in healthcare			0.81 (0.11)***
***R^2^***	**0.14**	**0.09**	**0.23**

Note: a. Robust standard errors are in parentheses. b. ** indicates significance at the 5% level, and *** indicates significance at the 1% level.

**Table 4.** Estimates of direct, indirect, and total effects from structural equation models (*N* = 313).

Variables	Without confounding factors		With confounding factors	
	Direct effect	Indirect effect	Direct effect	Indirect effect
Affordability: unable to get needed care because of costs	1.32 (0.82)	0.69**(0.33)	1.74**(0.80)	0.93**(0.41)
Negative experience with a provider	1.58**(0.70)	1.32***(0.29)	1.79**(0.71)	1.23***(0.32)

Note: Standard errors are in parentheses. Standard errors are computed based on 1000 Monte Carlo replications. ** indicates significance at the 5% level, and *** indicates significance at the 1% level.

## 5. Discussion

In this study, we examined the relationships between affordability (i.e., unable to get needed healthcare because of costs), negative experience with a healthcare provider, perceived racism, and healthcare system distrust among older Black American women. According to the results, women who reported affordability problems and a negative experience with a provider had higher levels of perceived racism and healthcare system distrust. Perceived racism also mediated the relationships between affordability, a negative experience with a provider, and healthcare system distrust. Sociodemographic characteristics, health status, and access to care factors were not identified as significant predictors of healthcare system distrust.

Affordability was a significant predictor of perceived racism and healthcare system distrust for older Black women. This is the first study to report these findings, which are of some importance. Affordability of care is not typically considered when assessing perceptions of racism [52] and healthcare system distrust. In a study exploring variations in perceived racial privilege and racial discrimination, Stepanikova and Oates [52] found that going without medical care due to cost was associated with higher perceived racial discrimination among all racial/ethnic groups, while among Whites, health insurance was associated with less perceived racial discrimination. There has been great emphasis on increasing health insurance coverage and reducing cost barriers to care [21]. Policies and programs, such as Medicaid Expansion and patient-centered medical home (PCMH) models, have been implemented to make health insurance more affordable for vulnerable populations, control rising healthcare costs, and enhance the patient experience of care [21]. In states where Medicaid expansion has been implemented, healthcare is more affordable, easier to access, and has improved health outcomes [53],[54]. However, the effect is inconsistent across racial/ethnic groups, and there is a lack of consensus on the impact of PCMH on racial/ethnic disparities [55],[56]. This is hardly surprising, given that disparities and racism are not always explicitly prioritized in every PCMH [57]. It is speculated that continued unexplained racial-ethnic differences in PCMH care may be due to differential healthcare and discriminatory experiences [58].

The association between negative experience with a healthcare provider and higher levels of perceived racism and distrust in the healthcare system supports similar findings from other studies [59],[60]. Healthcare providers are integral to the health encounter, and a negative experience with a provider can foster poor patient–provider relationships and contribute to adverse health outcomes [61]–[63]. It can be argued that a global measure of negative experiences with a provider captures a myriad of experiences that may or may not influence perceived racism and healthcare system distrust. However, according to a 2021 study by the Pew Research Center, 54% of Black women aged 50 and older reported having at least one of several negative experiences with healthcare providers in the past [16]. These experiences included having to speak up to get the proper care, feeling rushed by healthcare providers, being treated with less respect than other patients, feeling judged because of their weight, and having their health concerns not taken seriously [16]. Perceived racism during the medical encounter remains prevalent [60] and health outcomes remain dire for those who experience it. When individuals perceive discrimination in their healthcare interactions, they are less likely to engage in preventative health measures, such as cancer screenings and routine vaccinations [64]. The provider and patient encounter plays a key role in patient outcomes and remains an area that must be improved [61]. The scarcity of Black healthcare providers, who are often considered more likely to be culturally responsive to Black patients [65], may further exacerbate the impact of perceived racism and distrust on health outcomes among older Black women.

Our results suggest that perceived racism may be the pivotal factor driving older Black women's distrust of the healthcare system. Of note, perceptions of racism helped to explain all the statistically significant relationships observed with healthcare system distrust. Research shows that Black Americans experience discrimination in almost every aspect of their daily lives [31]. These experiences have far-reaching implications for Black Americans' well-being and social cohesion. Recent research indicates that six in ten Americans believe that racism against Black people is widespread and seven in ten Black Americans view discrimination as a major roadblock to progress for Black people in the US [25]. However, there is still reluctance to recognize racism as a root cause of racial health disparities [22]. This suggests that efforts to address distrust and ensure equitable access to care must go beyond the elimination of affordability or financial barriers to care [66].

This research has limitations. First, we used a convenient sample of women, which precludes generalizability to other Black American populations. Second, data are cross-sectional, so causal relationships cannot be established. Cross-sectional data may also provide biased estimates of mediation effects [67]. Third, data were self-reported and thus subject to recall and other types of response bias [68]. Fourth, our measures of affordability and negative experience with a provider are not comprehensive and may not capture all the nuances of the healthcare experience. Fourth, the data is not current. However, the issues of affordability, racism, and distrust are at the forefront of current national discourse [69],[70]. Research by the Commonwealth Fund documents the current impact and disparities in affordability, racism, and distrust among Black Americans [69],[71]. By examining data collected in previous years or decades, we can gain insights into how these issues have evolved, which can inform current discourse and efforts to address health equity among older Black women [72]. Finally, given the variability explained by the model, potentially important variables may be omitted in our analysis, which may change the observed relationships. Nonetheless, this study provides a unique look at the relationships between healthcare affordability, perceived racism and discrimination, and distrust of the healthcare system, which has not been addressed in the literature. It also serves to emphasize the persistent and elucidatory role of racism in the healthcare experiences of Black Americans. Our findings have significant implications for addressing systemic inequalities and building trust in the healthcare system.

The issues of affordability, perceived racism in healthcare, and healthcare system distrust negatively impact Black populations, especially older Black women. Health systems should be encouraged to actively combat racism and bias in all aspects of healthcare delivery, including expanding and diversifying the healthcare workforce at every level. Advocating for policy changes that provide comprehensive health insurance coverage and financial assistance will also be essential to improve access to care for older Black women. It is also imperative to conduct research and implement initiatives aimed at understanding and addressing these issues. Longitudinal studies tracking the healthcare experiences of older Black women and assessing the impact of interventions aimed at reducing issues including affordability, racism, and distrust are warranted. However, future work will still be hampered without acknowledging racial harm [73]. This would elevate the prioritization of these studies and programs and galvanize the accurate collection and availability of race and ethnicity data. Bias in existing data can result from excluding marginalized populations such as the older Black women in this study.

## 6. Conclusion

The findings of this research underscore the critical need to address the complex interplay between healthcare affordability problems, having a negative experience with a provider, and healthcare system distrust among older Black American women. The identification of perceived racism as a significant mediator emphasizes the pervasive influence of systemic biases in shaping healthcare experiences. To mitigate these disparities and foster trust, policymakers must prioritize interventions aimed at improving healthcare affordability, enhancing cultural competence among healthcare providers, and implementing anti-discrimination policies within healthcare settings. Additionally, targeted efforts to engage and empower older Black American women in healthcare decision-making processes are essential for fostering a more equitable and inclusive healthcare system.

## Use of AI tools declaration

The authors declare they have not used Artificial Intelligence (AI) tools in the creation of this article.


