# Supplementary material for: Affordability, negative experiences, perceived racism, and health care system distrust among black American women aged 45 and over
Source: AIMS Public Health. 2024 Sep 26;11(4):1030–48. doi: 10.3934/publichealth.2024053 (PMC11717543; doi:10.3934/publichealth.2024053)
Supplement: Supplementary file 1 [file publichealth-11-04-053-s001.pdf]

Public Health

AIMS Public Health, 11(4): 1030–1048.
DOI: 10.3934/publichealth.2024053
Received: 25 January 2024
Revised: 18 June 2024
Accepted: 02 July 2024
Published: 26 September 2024

https://www.aimspress.com/journal/aimsph

Research article

Affordability, negative experiences, perceived racism, and health care system distrust among black American women aged 45 and over

Jacqueline Wiltshire[1,*], Carla Jackie Sampson[2], Echu Liu[3], Myra Michelle DeBose[4], Paul I Musey, Jr.[5] and Keith Elder[6]

[1] Indiana University Fairbanks School of Public Health, Indianapolis, IN USA
[2] Robert F. Wagner Graduate School of Public Service, New York University, NY USA
[3] College for Public Health and Social Justice, Saint Louis University, MO USA
[4] College of Nursing and School of Allied Health, Northwestern State University, LA USA
[5] Indiana University School of Medicine, Indianapolis, IN USA
[6] Saint Xavier University, Chicago, IL USA

* **Correspondence**: Email: jcwiltsh@iu.edu.

Supplementary

Table S1. Health care system distrust scale responses (*N* = 313).

	Project	Strongly Agree (%)	Agree (%)	Not Sure (%)	Disagree (%)	Strongly Disagree (%)
a	Medical experiments can be done on me without my knowing about it.	7.4	18.5	17.2	28.8	28.1
b	My medical records are kept private.	25.9	41.8	19.8	7.7	4.8
c	People die every day because of mistakes by the health care system.	27.8	51.4	8.6	10.5	1.6
d	When they take my blood, they do tests they do not tell me about.	6.1	21.7	24.9	33.6	13.7
e	If a mistake were made in my health care, the health care system would try to hide it from me.	10.9	32.3	26.5	22.4	8.0
f	People can get access to my medical records without my approval.	5.4	28.4	18.2	29.0	18.9
g	The health care system cares more about holding costs down than it does about doing what is needed for my health.	18.2	41.2	12.8	19.8	8.0
h	I receive high quality medical care from the health care system.	16.6	57.5	11.2	12.8	1.9
i	The health care system puts my medical needs above all other considerations when treating my medical problems.	11.8	39.0	20.5	25.2	3.5
j	Some medicines have things in them that they do not tell you about.	11.8	44.4	13.7	22.7	7.4

Note: Items b, h, and j are reverse scored or coded to be consistent with the other items in the scale.

Items a, d, e, and j measure honesty. Items b and f measure confidentiality.

Items c and h measure competence. Items g and i measure fidelity.

Alpha reliability coefficient of scale = 0.78

Source: Armstrong K, McMurphy S, Dean LT, et al. (2008) Differences in the patterns of health care system distrust between blacks and whites. *J Gen Intern Med* 23: 827–833. https://doi.org/10.1007/s11606-008-0561-9

Table S2. Perception of racism in health care scale responses (*N* = 313).

Project	Strongly Agree (%)	Agree (%)	Not Sure (%)	Disagree (%)	Strongly Disagree (%)
Doctors treat African American and White people the same.	3.2	13.4	19.2	46.3	17.9
Racial discrimination in a doctor's office is common[a]	3.8	18.5	17.6	48.6	11.5
In most hospitals, African Americans and Whites receive the same kind of care.	2.9	20.5	17.3	41.2	18.2
African Americans can receive the care they want as equally as White people can.	2.9	25.6	12.1	40.9	18.5

Note: [a]Responses were reversed scored or coded to be consistent with the other items in the scale.

Alpha reliability coefficient of scale = 0.70.

Source: LaVeist, TA, Nickerson, KJ, Bowie, JV (2000) Attitudes about racism, medical mistrust, and satisfaction with care among African American and white cardiac patients. *Med Care Res Rev* 57: 146–161. https://doi.org/10.1177/1077558700057001S07

Table S3. Correlations among key study variables (*N* = 313)

**Measure/variables**	1	2	3	4
1. Distrust of the health care system				
2. Unable to get needed care because of costs	0.14*			
3. Perceived racism in health care system	0.43**	0.13*		
4. Negative experience with a health care provider	0.23**	0.05	0.27**	

Note: *$p < 0.05$. **$p < 0.001$.

© 2024 the Author(s), licensee AIMS Press. This is an open access article distributed under the terms of the Creative Commons Attribution License (https://creativecommons.org/licenses/by/4.0)

AIMS Press
